# Serological and molecular findings during SARS-CoV-2 infection: the first case study in Finland, January to February 2020

**DOI:** 10.2807/1560-7917.ES.2020.25.11.2000266

**Published:** 2020-03-19

**Authors:** Anu Haveri, Teemu Smura, Suvi Kuivanen, Pamela Österlund, Jussi Hepojoki, Niina Ikonen, Marjaana Pitkäpaasi, Soile Blomqvist, Esa Rönkkö, Anu Kantele, Tomas Strandin, Hannimari Kallio-Kokko, Laura Mannonen, Maija Lappalainen, Markku Broas, Miao Jiang, Lotta Siira, Mika Salminen, Taneli Puumalainen, Jussi Sane, Merit Melin, Olli Vapalahti, Carita Savolainen-Kopra

**Affiliations:** 1 Department of Health Security, Finnish Institute for Health and Welfare (THL), Helsinki, Finland; 2 University of Helsinki, Medicum, Department of Virology, Helsinki, Finland; 3 Institute of Veterinary Pathology, Vetsuisse Faculty, University of Zürich, Zürich, Switzerland; 4 Inflammation Center, Infectious Diseases, Helsinki University Hospital (HUSLAB) and University of Helsinki, Helsinki, Finland; 5 Department of Virology and Immunology, Helsinki University Hospital (HUSLAB) and University of Helsinki, Helsinki, Finland; 6 Infection-Hospital Hygiene Unit, Lapland Central Hospital, Rovaniemi, Finland; 7 Faculty of Biological and Environmental Sciences, University of Helsinki, Helsinki, Finland

**Keywords:** SARS-CoV-2, COVID-19, coronavirus, antibodies, humoral immunity, whole-genome sequencing, microneutralisation test, immunofluorescence assay, western blotting

## Abstract

The first case of coronavirus disease (COVID-19) in Finland was confirmed on 29 January 2020. No secondary cases were detected. We describe the clinical picture and laboratory findings 3–23 days since the first symptoms. The SARS-CoV-2/Finland/1/2020 virus strain was isolated, the genome showing a single nucleotide substitution to the reference strain from Wuhan. Neutralising antibody response appeared within 9 days along with specific IgM and IgG response, targeting particularly nucleocapsid and spike proteins.

On 31 December 2019, a cluster of pneumonia cases of unknown aetiology was reported in Wuhan, Hubei Province, China [1]. Severe acute respiratory syndrome coronavirus 2 (SARS-CoV-2) was isolated by Chinese scientists on 7 January 2020. To date, the SARS-CoV-2 virus causing the coronavirus disease (COVID-19) pandemic is spreading throughout the world.

Here we describe the timeline of events around the first COVID-19 case imported to Finland, and summarise the clinical, molecular and serological data. Successful SARS-CoV-2/Finland/1/2020 isolation enabled us to use the cytopathic effect (CPE)-based microneutralisation (MN) assay to detect SARS-CoV-2-specific neutralising antibody levels. Diagnostic serum samples of the case and three close contacts were analysed and compared with serum samples from the Finnish population collected in 2019.

## Clinical presentation and laboratory confirmation of the case

**Figure 1:**
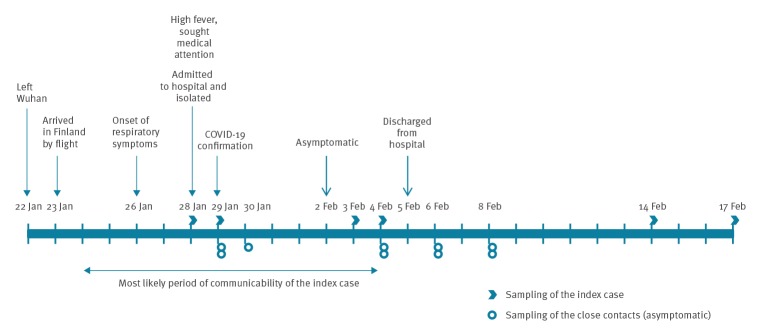
Timeline of events around the first COVID-19 case imported to Finland, January–February 2020

The first COVID-19 case in Finland was a female Chinese tourist in her 30s, who had left Wuhan on 22 January and arrived in Finland on 23 January. Her first symptoms were a runny nose on 26 January and nausea on 27 January. Because of high fever (39 °C), weakness and cough she sought medical attention on 28 January. Suspicion of COVID-19 led to her direct transfer to the Lapland Central Hospital in Rovaniemi, where she was isolated and sampled on 28 and 29 January for laboratory confirmation of SARS-CoV-2 infection (Figure 1). SARS-CoV-2 infection was confirmed from nasopharyngeal samples on 29 January by the Helsinki University Hospital Laboratory (HUSLAB), and further confirmed at the Finnish Institute for Health and Welfare (THL) (Table). Both laboratories performed real-time RT-PCR testing for three targets: the envelope (E), the RNA-dependent RNA polymerase (RdRp) and the nucleocapsid (N). Primers and probes were based on the Corman et al. method [2]. Cycle threshold (Ct) values above 37 were considered negative.

COVID-19: coronavirus disease.

**Table:** Laboratory data of the first case of SARS-CoV-2 infection, Finland, January–February 2020

Sampling day Day since the first symptoms	Specimen	PCR done at	E	RdRp	N	MN	IgM	IgG
**28 Jan 2020** Day 3	NPS	HUS THL	ND 30.49	ND 30.48	ND 31.59	NA	NA	NA
**29 Jan 2020** Day 4	NPA	HUS THL	31.18 27.13	27.56 28.43	28.29 28.73	NA	NA	NA
	NPS	HUS THL	28.15 29.59	27.13 30.87	28.82 31.78	NA	NA	NA
	Serum	THL UH	Neg Neg	Neg Neg	Neg Neg	< 4	< 20	< 20
**03 Feb 2020** Day 9	NPS	HUS THL	Neg Neg	Neg Neg	Neg Neg	NA	NA	NA
	Serum	UH	ND	Neg	Neg	60	80	80
**04 Feb 2020** Day 10	NPS	HUS THL	Neg Neg	Neg Neg	Neg Neg	NA	NA	NA
	Serum	ND	ND	ND	ND	72	160	160
**14 Feb 2020** Day 20	Serum	UH	Neg	Neg	Neg	160	320	1,280
**17 Feb 2020** Day 23	NPS	HUS THL	Neg Neg	Neg Neg	Neg Neg	NA	NA	NA

E: envelope protein gene; HUS: Helsinki University Hospital Laboratory; IgG: immunoglobulin G; IgM: immunoglobulin M; MN: microneutralisation test; N: nucleocapsid protein gene; NA: not applicable; ND: not done; Neg: negative; NPA: nasopharyngeal aspirate; NPS: nasopharyngeal swab; RdRp: RNA-dependent RNA polymerase gene; RT-PCR: reverse-transcription PCR; THL: Finnish Institute for Health and Welfare; UH: University of Helsinki.

The case had mild symptoms throughout the isolation period. She was tested PCR-negative in 3 and 4 February samples and, as considered asymptomatic, discharged from hospital on 5 February. One additional sample for serology and PCR was taken on 14 and 17 February, respectively.

Altogether 21 close contacts were identified of whom we could reach 17. Fourteen were still in Finland and placed in quarantine for 14 days. Information about three close contacts that had left the country was communicated to the competent authorities in their respective countries. For the remaining four close contacts, we had no contact details. Two of the 21 close contacts were closely co-exposed and therefore sampled on Days 4, 10, 12 and 14 after the first symptoms of the index case. Follow-up of all contacts ended on 11 February without secondary transmission events.

## SARS-CoV-2/Finland/1/2020 virus isolation

The SARS-CoV-2 virus SARS-CoV-2/Finland/1/2020 was isolated in a biosafety level 3 (BSL-3) laboratory in Vero E6 cells from the Day 4 nasopharyngeal swab (NPS) and nasopharyngeal aspirate (NPA) specimens (Table). The samples were inoculated into the cells for 1 h at 37 °C and 5% CO_2_ and fresh culture medium (Eagle's minimum essential medium (EMEM) supplemented with 2% fetal bovine serum (FBS), 0.6 μg/mL penicillin, 60 μg/mL streptomycin, 2 mM L-glutamine, 20 mM HEPES) were added for incubation. On the 4th day of incubation, half of the cultures were blind-passaged onto fresh Vero E6 cells and the rest of original passages were incubated further. After 4 days incubation a clear CPE was detected in the NPA-originated passage 2. The propagation of stock virus was done by passaging a low virus dose once again in Vero E6 cells, and virus culture was harvested on the 3rd day. Virus concentration was followed by RT-PCR. The Ct value for virus passage 1 on the 6th day of incubation was 17.65 and for passage 2 on the 2nd day, before any CPE was 20.63, whereas those of the NPS specimen remained at Ct values between 35 and 36.

## SARS-CoV-2/Finland/1/2020 whole-genome sequencing

Nearly the complete coding region of SARS-CoV-2 (GenBank accession number: MT020781) was sequenced from the NPS collected on Day 4 (Table) and the complete coding region was sequenced from the virus isolate obtained after three passages in Vero E6 cells. The virus had 1 nt substitution C21707T compared with the reference strain Wuhan-Hu-1 collected in Wuhan China, December 2019 (NC_045512) [3] which had led to a histidine to tyrosine (H49Y) substitution in the N-terminal domain of the spike glycoprotein.

### Antibody response during the SARS-CoV-2 infection

**Figure 2:**
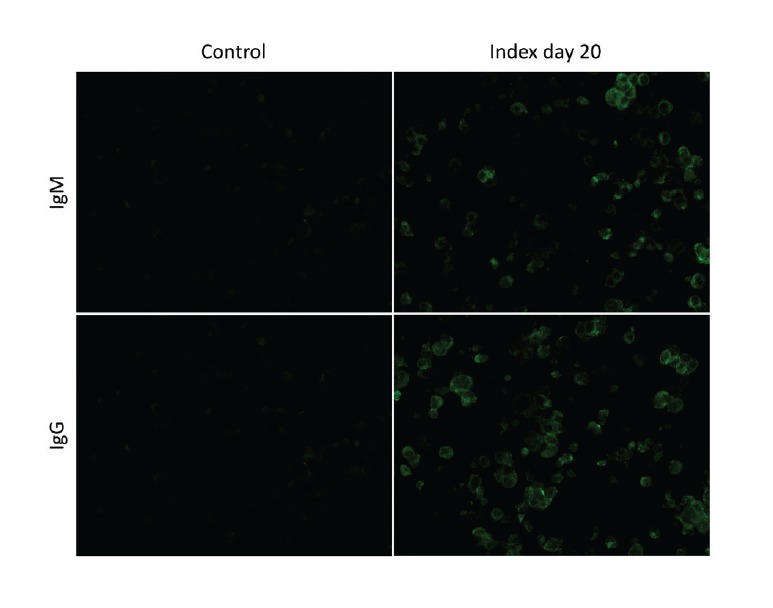
Immunofluorescence assay of serum samples, COVID-19 index case, Finland, January–February 2020

Serum samples were collected from the index case on Days 4, 9, 10 and 20 from onset of the first symptoms (Figure 1). Presence of serum IgM and IgG antibodies against SARS-CoV-2 was analysed by immunofluorescence assays (IFA) based on Vero E6 cells infected with passage 4 of the patient’s isolate SARS-CoV-2/Finland/1/2020 virus and transferred onto microscope slides and fixed with acetone (Figure 2). Serum samples from the index case were serially diluted and incubated for 2 h for IgM and 30 min for IgG. Antibodies were visualised with fluorescein isothiocyanate (FITC)-conjugated anti-human IgM or IgG antibodies. While the antibodies were undetectable on Day 4 after onset of symptoms, IgG titres rose to 80 and 1,280 and IgM titres to 80 and 320 on Days 9 and 20, respectively (Table). Random serum samples from staff members of the University of Helsinki (n = 19) did not show specific binding at dilutions greater than 20 (Figure 2).

COVID-19: coronavirus disease 2019.

Anti-SARS-CoV-2 IgM and IgG antibodies were detectable by immunofluorescence assay in samples from Days 9, 10 and 20 after onset of illness. Both IgM and IgG were found at a titre of 80 on Day 9, titres on Day 20 were 320 and 1,280. As an example, dilutions 1:20 and 1:160 from the Day 20 sample are shown for, respectively, IgM and IgG of the index case. Dilution 20 shown for the control serum.

**Figure 3:**
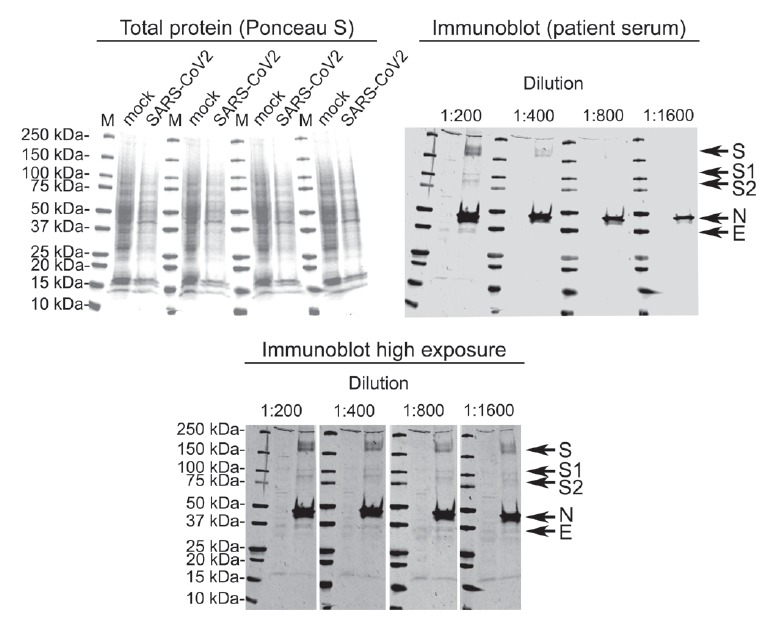
Western blot of mock- and SARS-CoV-2 infected Vero E6 cells using patient serum collected 20 days after onset of symptoms, Finland, January–February 2020

Mock- and SARS-CoV2-infected Vero E6 cells collected on Day 6 post infection were lysed in Laemmli sample buffer, and Western blotting (WB) of lysates was performed as described previously [4]. At 1:200 dilution, the convalescent serum on Day 20 identified SARS-CoV2 N, S and E protein bands (Figure 3). At higher exposure, all bands were detectable even at 1:1,600 serum dilution (Figure 3).

Top left panel: total protein staining (Ponceau S) of the nitrocellulose membrane before probing. Top right panel: strips probed with different dilutions of the patient serum at low exposure. Bottom panel: the same membranes individually contrasted for higher band intensity. The arrows indicate SARS-CoV-2 proteins, the labelling assumes that the migration of SARS-CoV-2 proteins was similar to that of Vero E6-expressed SARS-CoV proteins [23]. The bands migrating at ca 110 and 90 kDa probably represent S1 and S2, respectively. Marker M: Precision Plus Dual Colour Standards (Bio-Rad). The detection was done using Odyssey Infrared Imaging System (LI-COR) using goat anti-human IR800 conjugate at 1:10,000 dilution.

SARS-CoV-2-specific neutralising antibody levels were measured in duplicate with the MN test in a BSL-3 laboratory. The serum samples were heat-inactivated at 56 °C for 30 min and 2-fold serially diluted starting from 1:4 in EMEM supplemented with 2% of heat-inactivated FBS and antibiotics. Fifty plaque‐forming units (PFU) of the SARS-CoV-2/Finland/1/2020 strain were added to the serum dilutions and incubated for 1 h at 37 °C. Vero E6 cells (5 × 10^4^/well) were added to the virus–serum mix, and the mixture was incubated in 96-well plates for 4 days at 37 °C with 5% CO_2_. Neutralisation was assessed by CPE. The neutralisation endpoint was determined as the 50% endpoint of the serum that inhibited the SARS-CoV-2 infection observed by CPE of inoculated cells.

Diagnostic serum samples from the index case and her three asymptomatic close contacts were studied with the MN test. During the acute phase of infection, no neutralising antibodies were detected. The patient seroconverted for neutralising antibodies between Day 4 and 9, with the titre increasing to 160 on Day 20 (Table). The serum specimens were confirmed not to be toxic or infective to the cells as such.

Serum samples taken from the three close contacts tested negative in MN test. We also tested serum samples collected in 2019 from 83 Finnish subjects aged 4 to 89 years and all tested negative. Sera known to be positive for IgG against human coronavirus OC43 and 229E [5] and rabbit or guinea pig antibody against SARS-CoV N protein [6] could not neutralise the virus.

### Ethical statement

The investigations were carried out in accordance with the General Data Protection Regulation (Regulation (EU) 2016/679 and Directive 95/46/EC) and the Finnish Personal Data Act (Finlex 523/1999) The Finnish Communicable Diseases Act (Finlex 1227/2016) allows sampling for diagnostic and surveillance purposes.

The convalescent serum sample was obtained on 14 February through informed consent of the patient and research permits (TYH2018322, TYH2019263) from the Helsinki University Hospital Laboratory.

Finnish population serum samples were collected during 2019. The study protocol was approved by the Ethics Committee of the Department of Medicine, Helsinki University Hospital (Permission 433/13/03/00/15).

Serum samples of University of Helsinki staff members were used under informed consent.

## Discussion

In the early phase of the COVID-19 outbreak, confirmed cases outside China were mostly imported among travellers from Wuhan [7]. The first case in Finland was detected on 29 January among the first imported cases in Europe. The case presented mild symptoms without pneumonia: runny nose, nausea, high fever, cough, muscular weakness and fatigue. No secondary transmission events were detected despite active follow-up by the Lapland Hospital district and THL.

As at 17 March 2020, 358 additional laboratory-confirmed cases of COVID-19 have been detected in Finland. Many of them are travel-related (mostly from northern Italy and Austria) but there is also local transmission from the travel-related cases. The risk of widespread national community transmission of COVID-19 infection in the European Union, European Economic Area and the United Kingdom in the coming weeks is considered high by the European Centre for Disease Prevention and Control [8].

The sequence of the viral genome of the patient was nearly identical to the reference strain from Wuhan, reflecting an early importation from China. Later sequence information in Finland (up to 2 March) showed clustering with strains circulating in Italy (see nextstrain.org/ncov) [9].

Current guidelines from the World Health Organization for testing COVID-19 recommend collection of both acute and convalescent serum samples from patients for serological testing, which can support the identification of the immune response to a specific viral pathogen [10]. The SARS-CoV-2 nucleic acid has been found also in anal swabs and blood [11], however we did not detect it in serum samples in this case. As yet, only limited data are available on antibody responses during SARS-CoV-2 infection [11,12]. Further studies are needed to better understand the seroprevalence of antibodies to different corona viruses in populations and the role of these antibodies in the risk of disease.

In accordance with earlier findings [11], we found that both IgM and IgG titres were low or undetectable at on Day 4 (the second day after admission to hospital) yet increasing on Day 9–10, i.e. 5–6 days after the first sampling. Using other detection methods beyond IFA as well as recombinant antigens and analysing samples from a larger number of patients will shed more light on this. The time of first appearance of anti-SARS-CoV antibodies has ranged from Day 3 to 42 and Day 5 to 47 for IgM and IgG antibodies, respectively [13].

The WB of the serum sample collected at convalescence showed a prominent response against the N and S protein, confirming their role as main candidate diagnostic targets for antibody tests. However, the patient serum appeared to recognise also the E protein and the processed S1 and S2 proteins. Although WB detects mainly linear epitopes, the strong antibody response against the S protein correlated well with the results of the MN assay.

Monitoring of the binding antibodies is suggested to be a more sensitive method than measuring functional neutralising antibodies for serological detection of human coronavirus (hCoV) infections [14]. However, hCoV OC43 and 229E samples can also cross-react with SARS-CoV ELISA testing [15]. The SARS-CoV-2 CPE-based MN test using live virus appeared to be very specific, while laborious to conduct requiring a BSL-3 laboratory. An increase of at least 4-fold in the neutralising antibodies indicating a positive response was detected at Day 9–10 after the first symptoms and at Day 20, the antibody levels were still increasing. Our findings indicate that the MN assay is specific for functional SARS-CoV-2 antibodies and could be applied in surveillance of population immunity for this virus. The assay can be used as confirmatory tool for SARS-CoV-2 specificity in the development of more accessible diagnostic tools such as assays based on detecting binding antibodies. Previous studies on patients with SARS-CoV infection indicated that the median time for seroconversion was 20 days, by which time 60–75% of patients had IgG against the virus [13,16]. That IgM and IgG antibodies were present within 2 weeks from the onset of symptoms in our study suggests that early convalescent patients may be suitable sources of therapeutic antibodies [17]. In accordance with our finding, a recent preprint report on patients admitted to hospital with confirmed SARS-CoV-2 infection in China indicated that the median time to seroconversion was 11–14 days, depending on the immunological assay used [18].

No neutralising SARS-CoV-2 antibodies were detected in the close contacts nor in the control population samples collected during 2019 in Finland. A low prevalence (0.21%) of antibodies against Middle East respiratory syndrome coronavirus was reported in the general population of Qatar [19]. A meta-analysis of seroprevalence to SARS-CoV among different human populations yielded an overall low seroprevalence (0.10%), although it was slightly higher (0.23%) among healthcare workers and others who had close contact with SARS patients [20]. Binding and neutralising HCoV antibodies were found to be higher in older adults [14]. In total 97% and 99% of serum samples from healthy adults had antibodies to HCoV-229E and HCoV-OC43, respectively [21], and 75% and 65% of the children in the age group 2.5–3.5 years were found to be seropositive for, respectively, HCoV-NL63 and HCoV-229E [22].

While it has been suggested that the late seroconversion in most SARS patients reduces the value of serological assays during the incubation and initial phases of SARS [13], serological testing is suggested for the confirmation of a SARS CoV-2 infection [11]. After understanding better the kinetics, specificity and sensitivity of the assays in development, the serological testing may help contact tracing of clusters and have a role in diagnosing acute and past SARS-CoV-2 infections.

## References

[1] World Health Organization (WHO). Novel Coronavirus (2019-nCoV) Situation report - 1. Geneva: WHO, 21 Jan 2020. Available from: https://www.who.int/docs/default-source/coronaviruse/situation-reports/20200121-sitrep-1-2019-ncov.pdf

[2] Corman VM Landt O Kaiser M Molenkamp R Meijer A Chu DKW Detection of 2019 novel coronavirus (2019-nCoV) by real-time RT-PCR. Euro Surveill. 2020;25(3). 10.2807/1560-7917.ES.2020.25.3.2000045 31992387 PMC6988269

[3] Wu F Zhao S Yu B Chen YM Wang W Song ZG A new coronavirus associated with human respiratory disease in China. Nature. 2020;579(7798):265-9. 10.1038/s41586-020-2008-3 32015508 PMC7094943

[4] Korzyukov Y Hetzel U Kipar A Vapalahti O Hepojoki J Generation of anti-boa immunoglobulin antibodies for serodiagnostic applications, and their use to detect anti-reptarenavirus antibodies in boa constrictor. PLoS One. 2016;11(6):e0158417. 10.1371/journal.pone.0158417 27355360 PMC4927170

[5] Mäkelä MJ Puhakka T Ruuskanen O Leinonen M Saikku P Kimpimäki M Viruses and bacteria in the etiology of the common cold. J Clin Microbiol. 1998;36(2):539-42. 10.1128/JCM.36.2.539-542.1998 9466772 PMC104573

[6] Ziegler T Matikainen S Rönkkö E Osterlund P Sillanpää M Sirén J Severe acute respiratory syndrome coronavirus fails to activate cytokine-mediated innate immune responses in cultured human monocyte-derived dendritic cells. J Virol. 2005;79(21):13800-5. 10.1128/JVI.79.21.13800-13805.2005 16227300 PMC1262618

[7] Backer JA Klinkenberg D Wallinga J Incubation period of 2019 novel coronavirus (2019-nCoV) infections among travellers from Wuhan, China, 20-28 January 2020. Euro Surveill. 2020;25(5). 10.2807/1560-7917.ES.2020.25.5.2000062 32046819 PMC7014672

[8] European Centre for Disease Prevention and Control (ECDC). Novel coronavirus disease 2019 (COVID-19) pandemic: increased transmission in the EU/EEA and the UK – sixth update. Stockholm: ECDC, 12 Mar 2020. Available from: https://www.ecdc.europa.eu/en/publications-data/rapid-risk-assessment-novel-coronavirus-disease-2019-covid-19-pandemic-increased

[9] Hadfield J Megill C Bell SM Huddleston J Potter B Callender C Nextstrain: real-time tracking of pathogen evolution. Bioinformatics. 2018;34(23):4121-3. 10.1093/bioinformatics/bty407 29790939 PMC6247931

[10] World Health Organization (WHO). Laboratory testing for 2019 novel coronavirus (2019-nCoV) in suspected human cases. ISBN 978-92-4-000097-1. Geneva: WHO; 17 Jan 2020. Available from: https://www.google.com/url?sa=t&rct=j&q=&esrc=s&source=web&cd=1&ved=2ahUKEwiOw-i44ZLoAhWWFcAKHZmJCIoQFjAAegQIBBAB&url=https%3A%2F%2Fapps.who.int%2Firis%2Frest%2Fbitstreams%2F1266309%2Fretrieve&usg=AOvVaw1YNVgNwua9Dpj5c-PSD5c8

[11] Zhang W Du RH Li B Zheng XS Yang XL Hu B Molecular and serological investigation of 2019-nCoV infected patients: implication of multiple shedding routes. Emerg Microbes Infect. 2020;9(1):386-9. 10.1080/22221751.2020.1729071 32065057 PMC7048229

[12] Bai SL Wang JY Zhou YQ Yu DS Gao XM Li LL [Analysis of the first cluster of cases in a family of novel coronavirus pneumonia in Gansu Province]. Zhonghua Yu Fang Yi Xue Za Zhi. 2020;54(0):E005. Chinese. 32064855 10.3760/cma.j.issn.0253-9624.2020.0005

[13] Chen X Zhou B Li M Liang X Wang H Yang G Serology of severe acute respiratory syndrome: implications for surveillance and outcome. J Infect Dis. 2004;189(7):1158-63. 10.1086/380397 15031782 PMC7110198

[14] Gorse GJ Donovan MM Patel GB Antibodies to coronaviruses are higher in older compared with younger adults and binding antibodies are more sensitive than neutralizing antibodies in identifying coronavirus-associated illnesses. J Med Virol. 2020;92(5):512-7. 10.1002/jmv.25715 32073157 PMC7166442

[15] Woo PC Lau SK Wong BH Chan KH Hui WT Kwan GS False-positive results in a recombinant severe acute respiratory syndrome-associated coronavirus (SARS-CoV) nucleocapsid enzyme-linked immunosorbent assay due to HCoV-OC43 and HCoV-229E rectified by Western blotting with recombinant SARS-CoV spike polypeptide. J Clin Microbiol. 2004;42(12):5885-8. 10.1128/JCM.42.12.5885-5888.2004 15583332 PMC535232

[16] Peiris JSM Chu CM Cheng VCC Chan KS Hung IFN Poon LLM Clinical progression and viral load in a community outbreak of coronavirus-associated SARS pneumonia: a prospective study. Lancet. 2003;361(9371):1767-72. 10.1016/S0140-6736(03)13412-5 12781535 PMC7112410

[17] Chen L Xiong J Bao L Yuan S Convalescent plasma as a potential therapy for COVID-19. The Lancet Infectious Diseases. Available online 27 February 2020. Forthcoming 10.1016/S1473-3099(20)30141-9 PMC7128218 32113510

[18] Zhao J, Yuan Q, Wang H, Liu W, Liao X, Su Y et al. Antibody responses to SARS-CoV-2 in patients of novel coronavirus disease. Pre-print. 2019medRxiv 2020.03.02.20030189. 10.1093/cid/ciaa344 PMC7184337 32221519

[19] Al Kahlout RA Nasrallah GK Farag EA Wang L Lattwein E Müller MA Comparative serological study for the prevalence of anti-MERS coronavirus antibodies in high- and low-risk groups in Qatar. J Immunol Res. 2019;2019:1386740. 10.1155/2019/1386740 30906787 PMC6398027

[20] Leung GM Lim WW Ho LM Lam TH Ghani AC Donnelly CA Seroprevalence of IgG antibodies to SARS-coronavirus in asymptomatic or subclinical population groups. Epidemiol Infect. 2006;134(2):211-21. 10.1017/S0950268805004826 16490123 PMC2870380

[21] Che XY Qiu LW Liao ZY Wang YD Wen K Pan YX Antigenic cross-reactivity between severe acute respiratory syndrome-associated coronavirus and human coronaviruses 229E and OC43. J Infect Dis. 2005;191(12):2033-7. 10.1086/430355 15897988 PMC7109809

[22] Dijkman R Jebbink MF El Idrissi NB Pyrc K Müller MA Kuijpers TW Human coronavirus NL63 and 229E seroconversion in children. J Clin Microbiol. 2008;46(7):2368-73. 10.1128/JCM.00533-08 18495857 PMC2446899

[23] Wu XD Shang B Yang RF Yu H Ma ZH Shen X The spike protein of severe acute respiratory syndrome (SARS) is cleaved in virus infected Vero-E6 cells. Cell Res. 2004;14(5):400-6. 10.1038/sj.cr.7290240 15450134 PMC7091875

